# Case report: Complete pathologic response with first-line immunotherapy combination in a young adult with massive liver dissemination of mismatch repair–deficient metastatic colorectal cancer: Immunological and molecular profiling

**DOI:** 10.3389/fonc.2022.964219

**Published:** 2022-12-08

**Authors:** Francesca Bergamo, Silvia Dalla Santa, Fotios Loupakis, Krisida Cerma, Anna Tosi, Caterina De Grandis, Anna Dalla Pietà, Enrico Gringeri, Valentina Angerilli, Gaetano Ramondo, Alessandro Rago, Fabiola Cecchi, Stephen Benz, Umberto Cillo, Angelo Paolo Dei Tos, Vittorina Zagonel, Matteo Fassan, Antonio Rosato, Sara Lonardi

**Affiliations:** ^1^ Oncology Unit 1, Department of Oncology, Veneto Institute of Oncology IOV - IRCCS, Padua, Italy; ^2^ Department of Surgery, Oncology and Gastroenterology, University of Padua, Padua, Italy; ^3^ Immunology and Molecular Oncology Diagnostics Unit, Veneto Institute of Oncology IOV - IRCCS, Padua, Italy; ^4^ Hepatobiliary Surgery and Liver Transplantation Unit, Department of Surgery, Oncology and Gastroenterology, Padua University Hospital, Padua, Italy; ^5^ Surgical Pathology Unit, Department of Medicine (DIMED), University of Padua, Padua, Italy; ^6^ Radiology Unit, Veneto Institute of Oncology IOV - IRCCS, Padua, Italy; ^7^ NantOmics, Rockville, MD, United States; ^8^ Veneto Institute of Oncology IOV - IRCCS, Padua, Italy; ^9^ Oncology Unit 3, Department of Oncology, Veneto Institute of Oncology IOV - IRCCS, Padua, Italy

**Keywords:** colorectal cancer, nivolumab, ipilimumab, MSI-H, complete pathological response

## Abstract

The current level of evidence for immunotherapy in previously untreated microsatellite unstable metastatic colorectal cancer is based on recent pieces of evidence of few studies that demonstrated durable response and clinical benefit, in terms of objective response rate, disease control rate, and progression-free survival in this subgroup of patients. On the basis of combinatorial immunotherapy with nivolumab plus ipilimumab, we report the exceptional case of a complete pathological response in a 21-year-old woman presenting a clinically aggressive stage IV colorectal cancer with massive nodal and liver involvement. Extensive molecular analyses based on whole genome next-generation DNA sequencing, RNA sequencing, fluorescent multiplex immunohistochemistry, and flow cytometry provided a detailed description of tumoral and immunological characteristics of this noteworthy clinical case.

## Introduction

Colorectal cancer (CRC) is the fourth cause of cancer-related deaths worldwide (1). Deficient mismatch repair/microsatellite instability-high (dMMR/MSI-H) occurs in about 5% of metastatic CRC (mCRC) (2). The optimal first-line treatment regimen in mCRC has been represented by combining chemotherapy and anti-vascular endothelial growth factor or anti-epidermal growth factor receptor antibodies. However, its clinical benefit is limited with a median overall survival (OS) of approximately 30 months (3, 4). In the last 2 years, practice-changing results of two studies, KEYNOTE-177 and CHECKMATE-142, validated immunotherapy in previously untreated microsatellite unstable (dMMR/MSI-H) mCRC. The first trial demonstrated durable clinical benefits of pembrolizumab in terms of progression-free survival (PFS) compared with standard chemotherapy in patients with dMMR/MSI-H mCRC (16.5 months vs. 8.2 months; HR, 0.59; 95% CI, 0.45 to 0.79) and a significant increase in objective response rate (ORR). The second study confirmed the benefits of immunotherapy in this subgroup and identified a synergy from the dual blockade of Programmed cell death-1 PD-1 (PD-1) and Cytotoxic T-Lymphocyte Antigen 4 (CTLA-4), and nivolumab and ipilimumab. The primary endpoint ORR was 69% (95% CI, 53 to 82) with a disease control rate (DCR) of 84% (95% CI, 70.5 to 93.5) and a radiological complete response rate of 13% at median follow-up of 29.0 months (5–7).

Here, we describe an exceptional case of a clinically complex MSI-H stage IV CRC associated with Lynch syndrome. The patient received exclusively nivolumab plus ipilimumab as upfront treatment and obtained an impressive response. She underwent surgical resection of radiological residual disease with the confirmation of a pathological complete response (pCR), a very rare event with standard chemotherapies (8).

## Case report

**Figure 1:**
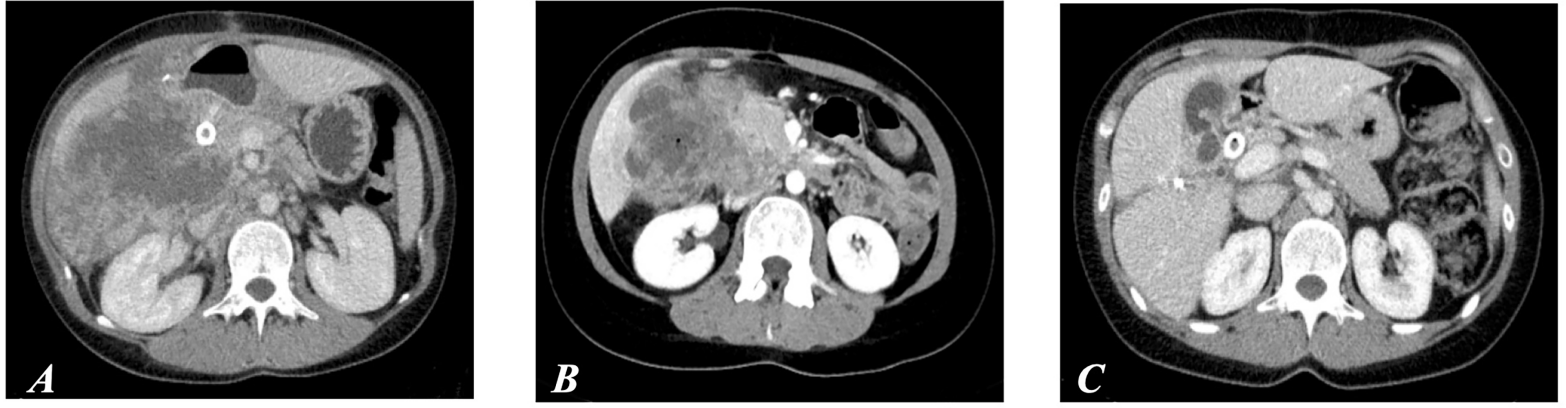
**(A)** CT scan performed before surgery on primary tumor. **(B)** CT at day 42, after the first cycle of immunotherapy. **(C)** CT after six cycles of immunotherapy (first evidence of partial response according to RECIST 1.1 criteria).

In March 2017, a 21-year-old woman with no significant past medical or familiar history was referred to a Community Hospital after a 3-month history of diarrhea, progressive worsening of asthenia (up to grade 3), and 13% body weight loss in 8 weeks. Visit and laboratory tests showed clinical deterioration [Eastern Cooperative Oncology Group (ECOG) Performance Status (PS) = 3], hyperpyrexia, increase in white blood count, and thrombocytosis. Abdomen computed tomography (CT) and magnetic resonance imaging (MRI) showed a large neoplastic mass involving the ascending colon, the right colic flexure (maximum diameter of 13 cm) and the liver, suspected infiltration of the head of the pancreas and the duodenum, extensive abdominal lymphadenopathies, and ascites. Moreover, colonic perforation was suspected due to perilesional fluid collection (**Figure 1A**).

**Figure 2:**
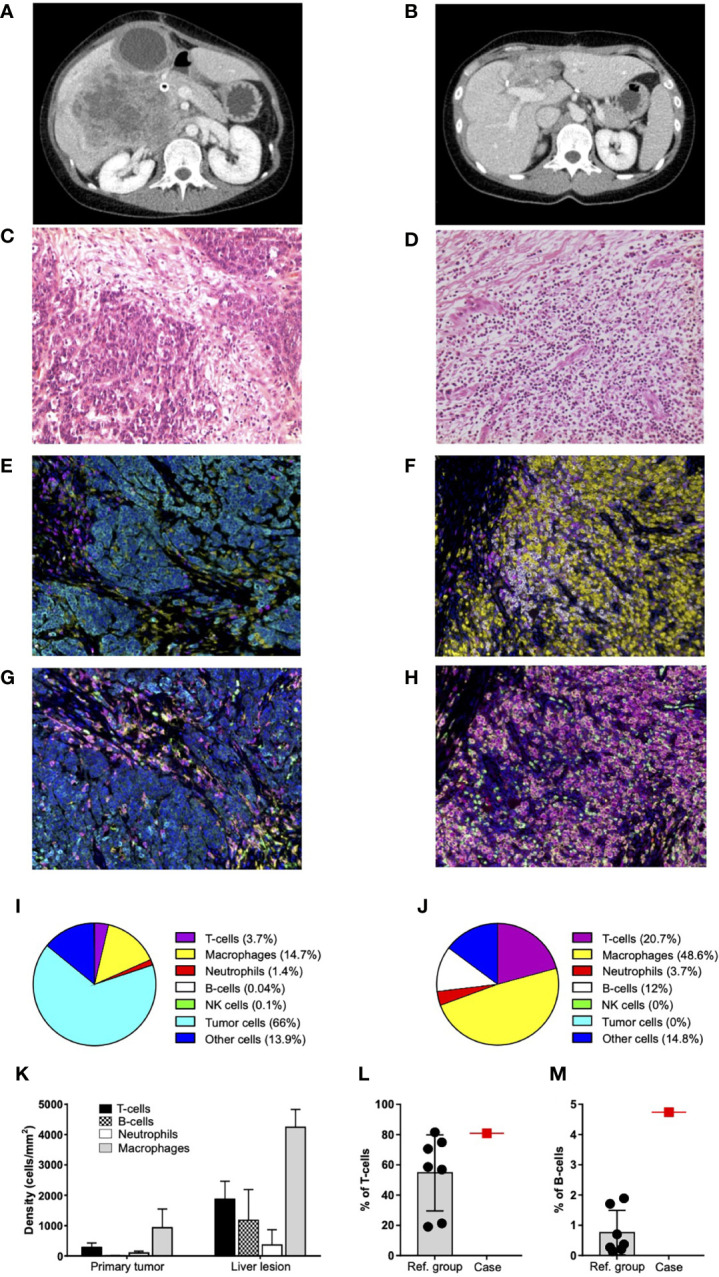
Characterization of primary tumor and liver lesion. **(A)** Re-staging scan before treatment start: large liver metastases in the right lobe associated with a wide abscess in S4. **(B)** Re-staging CT scan after nine cycles of immunotherapy and surgical resection of residual disease: outcomes of hepatic surgery without any evidence of residual disease. **(C)** Primary tumor from colonic resection: poorly differentiated adenocarcinoma (hematoxylin and eosin stain at ×200 magnification on light microscopy). **(D)** Liver lesion after nine cycles of immunotherapy and surgical resection: fibrous scar tissue and flogosis, without any evidence of residual neoplastic disease (hematoxylin and eosin stain at ×200 magnification on light microscopy). **(E, F)** Representative seven-color mIHC images of immune cell infiltrates in PT **(E)** and LL **(F)**; staining antibodies of “panel 1”: CD3 (magenta), CD68 (yellow), CD20 (white), CD56 (green), neutrophil elastase (red), pan-cytokeratin (cyan), and nuclei (blue). Original magnification, ×20. **(G, H)** Representative seven-color mIHC images of myeloid composition of PT **(G)** and LL **(H)**; staining antibodies of “panel 2”: CD68 (magenta), CD11c (red), CD11b (green), CD163 (yellow), neutrophil elastase (white), pan-cytokeratin (cyan), and nuclei (blue). Original magnification, ×20. **(I, J)** Pie charts of cumulative mIHC data for PT **(I)** and LL **(J)**. Data reported for each cell subset are the mean values of 10 fields from the same section. The total number of cells analyzed in PT **(I)** and LL **(J)** is 24,272 and 35,141, respectively. T cells, macrophages, neutrophils, B cells, natural killer (NK) cells, and tumor cells are identified as CD3+, CD68+, neutrophil elastase+, CD20+, CD56+, and pan-cytokeratin+ cells, respectively; “other cells” refer to cells negative for all these markers. **(K)** mIHC cell densities (cells/mm^2^) of immune cells infiltrating PT and LL. Mean values and SD derived from the analysis of the same 10 fields considered in **(I, J)** pie charts. **(L, M)** Percentages of T cells (CD3+) **(L)** and B cells (CD20+) **(M)** infiltrating fresh liver lesions, as assessed by flow cytometry. Patient data (case; red square) are reported together with those from the reference group (Ref. group; black dots). Data are gated on total leukocytes (CD45+). Mean and SD of the reference group are reported.

The patient underwent an urgent right colonic resection. Histology evidenced a poorly differentiated colonic adenocarcinoma (CRC), pT4a N2b based on TNM classification with macroscopic residual of disease (**Figure 2C**). The carcinoembrionyc antigen (CEA) level was determined at the diagnosis and subsequently at CT scan evaluations; it remained within normal limits during all the history of the disease.

DNA sequencing, performed subsequently at our center by mass spectrometry (with Kit Myriapod Colon status, Diatech Pharmacogenetics – MassArray analyzer), and immunohistochemistry (IHC) revealed a RAS/BRAF wild-type status, a high tumor mutational burden (TMB), with more than 1,700 somatic non-synonymous variants, and an estimated exonic mutation rate of 68.3 mutations/megabase.

The determination of microsatellite instability was evaluated by multiplex amplification with fluorescent primers and subsequent DNA fragment analysis on automated sequencer (Titano MSI kit CE-IVD Diatech Pharmacogenetics). It confirmed a germline non-sense mutation in MLH1 gene with a complete loss expression of MLH1 and PMS2 (mismatch repair deficiency), and Lynch syndrome was diagnosed.

RNA sequencing (RNAseq) data were used to assess potential overexpression of key immune modulators and also to assign the patient to one of the four colorectal consensus molecular subtypes (CMSs). The patient’s CMS was predicted as CMS4 (9), whereas RNAseq of immunotherapy-related genes revealed a high expression of Indoleamine 2,3-Dioxygenase 1 (IDO1) only.

The post-operative course was complicated by jaundice and hyperbilirubinemia (up to 8 mg/dl); hepatic ultrasound showed bile duct dilatation due to duodenal compression, which required insertion of a biliary metallic stent. After 28 days, she was dismissed with a mild improvement of general conditions and referred to our Comprehensive Cancer Center. Two days later, worsening of clinical conditions, hyperpyrexia (up to 39°C), and persistent alteration of liver function required immediate hospitalization. A re-staging CT scan confirmed large liver metastases, with diameter up to 12.2 cm, associated to hilar and abdominal lymphadenopathy, and a 7-cm abscess associated with cancer in S4 (**Figure 2A**).

An endoscopic retrograde cholangiopancreatography evidenced the partial obstruction of the biliary metallic stent, which required the insertion of an additional self-expandable metallic stent. Although liver function progressively improved, clinical conditions remained extremely unstable with recurrent febrile episodes, elevation of inflammatory markers, and a high risk of septic shock.

A multidisciplinary team extensively discussed the options of: i) a surgical exploration, ii) standard systemic treatment, or iii) opportunity for molecularly driven clinical trial.

Over a 48-h observation period, fever resolved and general conditions and liver functions improved, and she was enrolled in a phase 2 clinical trial (5) and received nivolumab (3 mg/kg q2w) plus ipilimumab (1 mg/kg q6w).

On 28 April 2017, nivolumab plus ipilimumab treatment was started. After 2 h from the administration, the patient had a progressive pain worsening in right hypochondrium and a sudden drop of hemoglobin. An ultrasound revealed bleeding of liver metastases requiring radiological embolization. After a few hours, the patient had hyperpyrexia and shivering, increase of inflammation markers, and hypotension. On 30 April 2017, the patient was transferred to the intensive care unit; blood cultures grew *Enterococcus faecium*, and meropenem and daptomycin were started, with a rapid general improvement and defervescence. At day 14 from the first administration, the patient received the second planned dose. A follow-up ultrasound showed a major shrinkage of the hepatic abscess. Thus, she was discharged, and the following cycles were planned as an outpatient.

General conditions rapidly improved over the next 4 weeks. A CT scan after the first cycle showed an initial reduction of all target lesions (**Figure 1B**). A massive shrinkage and the best response were achieved after six cycles, being maintained thereafter up to the completion of 9 cycles over a 12-month period, without any toxicity or delay (**Figures 1C**, **2B**).

On the basis of clinical course and multidisciplinary discussion, a surgical abdominal exploration was decided without evidence of extrahepatic involvement, whereas an intraoperative ultrasound suspected S4b-S5 metastatic residual disease. Consequently, an anatomic liver resection of *en bloc* S4b-S5, cholecystectomy, and hilar lymphadenectomy were performed. The post-operative course was uneventful, and the patient was discharged in post-operative day 6.

Liver lesion (LL) histology evidenced a pCR with fibrous tissue, necrosis and lympho-histiocytic inflammation, and no residual tumor cells (**Figure 2D**). Sample was graded as tumor regression score equal to 1 (10, 11).

Fluorescent multiplex IHC (mIHC) was performed to characterize lymphocyte population and immune checkpoint expression. mIHC analysis was carried out on of formalin-fixed paraffin-embedded tissue samples from primary tumor (PT) and secondary LL, whose cell composition is described in **Figures 2E–J**. According to histology, no cytokeratin+ tumor cells were detected in LL (**Figures 2F, H, J**), whereas infiltrating immune cells were increased at least five-folds compared to PT (**Figures 2I–K**). In particular, B cells increased more than 200-folds (**Figures 2I–K**) and appeared organized in agglomerates resembling tertiary lymphoid structures (**Supplementary Figure S1**). Flow cytometry analysis of total leukocytes infiltrating the fresh lesion revealed that the percentages of T- and B cells were higher than in the reference group (**Figures 2L, M**, **S2**), which consisted of liver metastases (n=7) collected from additional CRC patients treated with standard chemotherapy (**Supplementary Table 1**). Moreover, peripheral blood T and B cells increased after treatment start (**Supplementary Figure S3**).

**Figure 3:**
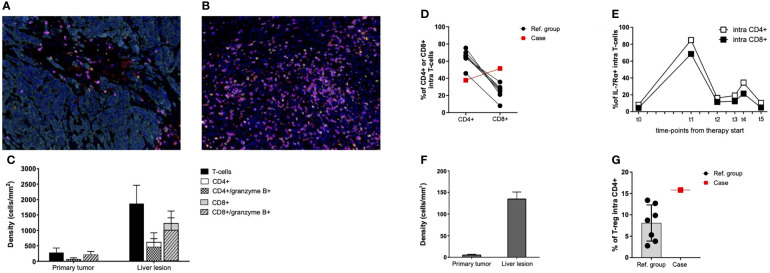
T-cell functional state in primary tumor, liver lesion, and PBMCs. **(A, B)** Representative seven-color mIHC images of T-cell infiltrates of PT **(A)** and LL **(B)**; staining antibodies of “panel 3”: CD3 (pink), CD8 (magenta), CD4 (red), granzyme B (yellow), FoxP3 (green), pan-cytokeratin (cyan), and nuclei (blue). Original magnification, ×20. **(C)** mIHC cell densities of CD4+, CD8+, and granzyme B+ T cells in 10 fields of PT and LL samples. Mean values and SD are reported for each cell population. **(D)** Percentages of CD4+ and CD8+ T cells infiltrating fresh liver lesions from the patient (Case; red square) and from the reference group (Ref. group; black dots), as assessed by flow cytometry. Data are gated on CD3+ cells. Connected date derived from the same lesion. **(E)** Percentages of IL-7R–expressing circulating T cells before therapy start (t0) and during treatment (t1 = disease re-staging after four cycles of immunotherapy, t2 = disease re-staging after six cycles of immunotherapy, t3 = disease re-staging after eight cycles of immunotherapy, and t4 = before surgical resection of the liver residual disease), as assessed by flow cytometry. Data are gated on CD4+ and CD8+ T cells before the start of immunotherapy. **(F)** mIHC cell densities of T-regulatory cells (CD3+/CD4+/FoxP3+) detected in PT and LL samples. Mean values and SD are reported. Data derived from the same 10 fields previously considered **(C)**. **(G)** Percentages of T-regulatory cells (CD25bright/CD127low) infiltrating fresh liver lesions from the patient (Case; red square) and from the reference group (Ref. group; black dots), as assessed by flow cytometry. Data are gated on CD4+ T cells.

A deeper assessment of lymphocyte populations disclosed a strong prevalence of CD8+ T cells in PT (CD8+/CD4+ ratio = 3.2) that was partially maintained in LL (CD8+/CD4+ ratio = 2) (**Figures 3A–C**). These data were confirmed in the fresh sample (CD8+/CD4+ ratio = 1.4) and completely differed from the reference group (CD8+/CD4+ ratio = 0.38; **Figure 3D**). Notably, in the PT, almost all T cells stained positive for granzyme B, irrespective of the subset phenotype (**Figures 3A, C**). This activation status persisted in LL (**Figures 3B, C**), where most of infiltrating T cells showed an effector-memory phenotype with a minority being terminally differentiated (**Supplementary Figure S4**). Peripheral blood T cells disclosed an expression peak of the IL-7 receptor (IL-7Rα) just after treatment start (**Figure 3E**), suggesting the induction of a memory pool subsequently observed in the LL. T-regulatory cells were already detected in PT and increased 20-folds in LL (**Figure 3F**). They were higher than in the reference group (**Figure 3G**).

**Figure 4:**
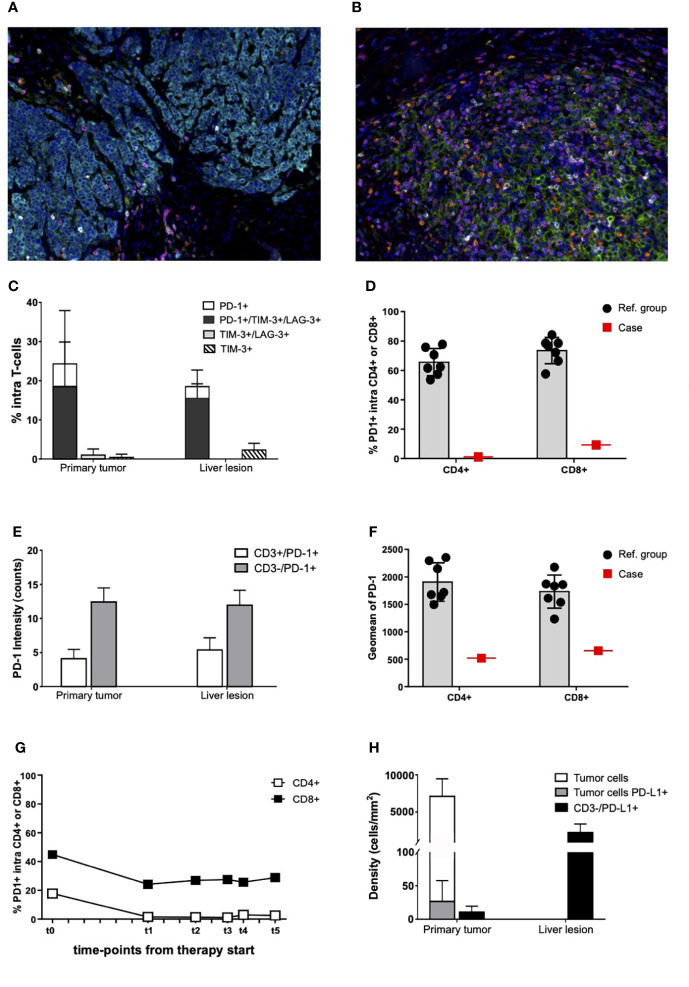
Immune checkpoint expression in primary tumor, liver lesion, and PBMCs. **(A, B)** Representative seven-color mIHC images of immune checkpoint expression in PT **(A)** and in LL **(B)**; staining antibodies of “panel 4”: CD3 (magenta), PD-1 (white), PD-L1 (green), TIM-3 (yellow), LAG-3 (red), pan-cytokeratin (cyan), and nuclei (blue). Original magnification, ×20. **(C)** Percentages of PD-1–, TIM-3–, and LAG-3–expressing T cells on total CD3+ cells infiltrating the PT and the LL, as assessed by mIHC. Mean values and SD are reported for each cell population. Data derived from analysis of 10 fields of each sample. **(D)** Percentages of PD1+ cells infiltrating fresh liver lesions from the patient (Case; red square) and from the reference group (Ref. group; black dots), as assessed by flow cytometry. Data are gated on CD4+ and CD8+ T cells. **(E)** mIHC analysis of PD-1 expression intensity on PD-1+ T cells (CD3+) and other PD-1+ cells (PD-1+/CD3−) infiltrating the PT and the LL. Mean values and SD are reported for each cell population. Data derived from analysis of the same 10 fields previously considered **(C)**. Intensity values are expressed in counts. **(F)** Flow cytometry data of PD-1 expression intensity (Geomean) on PD-1+ cells infiltrating fresh liver lesions from the patient (Case; red square) and the reference group (Ref. group; black dots). Data are gated on CD4+ and CD8+ T cells. **(G)** Percentages of PD-1–expressing circulation T cells before therapy start (t0) and during treatment (t1 = disease re-staging after four cycles of immunotherapy, t2 = disease re-staging after six cycles of immunotherapy, t3 = disease re-staging after eight cycles of immunotherapy, and t4 = before surgical resection of the liver residual disease), as assessed by flow cytometry. Data are gated CD+ and CD8+ cells. **(H)** mIHC cell densities of PD-L1–expressing cells in PT and LL. Mean values and SD are reported for each cell population. Data derived from analysis of the same 10 fields previously considered **(C)**.

Moreover, mIHC showed that 25% of T cells expressed PD-1 in PT, most of them being also TIM-3+ and LAG-3+ (**Figures 4A, C**). These percentages remained essentially unchanged in LL after treatment (**Figures 4B, C**). Cytometry data of LL additionally demonstrated that the percentage of PD-1+ cells within either CD4+ or CD8+ populations was much lower than in the reference group (**Figure 4D**). In both PT and LL, intensity of PD-1 expression in T cells was low (**Supplementary Figure S5** and **Figure 4E**) and differed clearly from the reference group (**Figure 4F**). PD-1 expression was appreciable in peripheral blood T cells before treatment and decreased after immunotherapy start (**Figure 4G**). A small fraction of cancer cells expressed PD-L1 in PT (**Figures 4A, H**). Putative PD-L1+ macrophages were organized in rare agglomerates in the PT, whereas they were scattered and highly increased in LL (**Supplementary Figure S6** and **Figures 4B, H**).

At the time of writing, 4 years since surgery, the patient is in excellent clinical conditions. She received immunotherapy until the end of 2018. A re-staging CT scan was performed every 8 weeks for 2 years and then every 16 weeks without evidence of disease recurrence. She lives a completely normal life without significant consequences related to surgery or immunotherapy. Regular oncologic follow-up and Lynch syndrome surveillance are still ongoing.

## Discussion

In this case report, we describe the case of a very young patient with a Lynch syndrome with MSI-H stage IV mCRC. The patient received exclusively nivolumab plus ipilimumab as upfront treatment within a phase 2 study and obtained an impressive and durable response, in line with clinical data of the trial CheckMate-142 (6, 7) and particularly with results obtained in a previously untreated patient’ cohort receiving immunotherapy combination (5).

In this regard, Chalabi et al. presented a study with a single-dose of neoadjuvant ipilimumab plus nivolumab in early-stage CRC, resulting in 100% major pathological response and 57% pCR in a total of seven MSI-H tumors (12). Moreover, very recently, Cercek and colleagues reported that single check point blockade in mismatch repair–deficient II or III rectal cancer reached 100% of clinical response rate (13). Although the sample size and the follow-up are limited, this amazing result and the growing pieces of evidence confirm the high efficacy of immunotherapy as upfront therapy and support its role even in the neoadjuvant setting.

From a clinical perspective, this case remarks the following open issues: a) whether immunotherapy should be always offered upfront to all patients with MSI-H; b) which is the optimal treatment duration; c) how to properly interpret radiological data, d) which could be reliable surrogate markers of a pCR; and therefore e) whether surgery on residual disease is really necessary.

To date, a large part of patient with advanced MSI-H mCRC are not candidate to radical surgery, and data about pathological response are limited. Our case provided a rare opportunity for running a parallel extensive translational study on primary and post-treatment samples. MSI-H status and high TMB support the high immunogenic features of this tumor and predicted sensitivity to immunotherapy (14, 15). On the other hand, a high expression of IDO1 and CMS4 subtype is somewhat unexpected, with these features being advocated as putative mechanisms of immune resistance (16). Moreover, CMS4 is not typical of MSI-H and BRAF wild-type tumors, which normally belong to CMS1 (9). Only a fraction of MSI-H CRCs is associated to Lynch syndrome, but preliminary data on their response to immunotherapy are based on small subgroup analyses and do not definitely clarify whether they benefit more than sporadic tumors (5, 6).

Tumor neoantigens generated by the high TMB likely induced a strong immune response even before immunotherapy start. Indeed, PT disclosed a strong prevalence of CD8+ over CD4+ T cells with both subsets having an activated and cytotoxic phenotype. Such infiltrating immune cells largely increased in post-treatment LL as compared to PT, with a partially maintained CD8+/CD4+ ratio but the acquisition of an effector-memory phenotype. With regard to B cells, they not only strongly increased in LL but organized in agglomerates resembling tertiary lymphoid structures.

Finally, also, T-regulatory cells, already detected in PT, increased in LL.

All these features are strongly reminiscent for a powerful boost of a pre-existing immune response. Accordingly, kinetics analysis of circulating cells confirmed that immunotherapy unleashed the patient’s immune response, especially during the first 6–9 months of therapy.

In fact, the raising of T and B cells and the rapid increase of IL-7Rα-expressing T cells indicated the development and the maintenance of memory pools (17, 18) and anticipated the immune features found in the LL.

The fact that T and B cells were more represented in LL together with a cytotoxic phenotype of both CD4+ and CD8+ cells and a prevalence of CD8+ T-cells are all features associated with a better prognosis in CRC (19–21). Furthermore, the presence of tertiary lymphoid structures is another marker associated with a favorable clinical prognosis not only in CRC (22–24). Notwithstanding, the CD4+ and CD8+ T cells infiltrating the LL appeared poorly proliferating and had a moderate expression of IL-7Rα (data not shown), whose downregulation prepares cells for death during the resolution of the immune response (25). In the LL, we also observed more suppressive Treg and PD-L1+ macrophages. Collectively, these data are not only the signature of an immune response that is still operative and specialized, but also the mark of resolution after tumor eradication.

Regarding immune checkpoint expression, the percentage of T cells expressing PD-1 in PT remained essentially unchanged in LL after treatment, and this, in turn, appeared lower than in the reference group. Moreover, PD-1 was present at a low intensity on infiltrating T cells, and its expression decreased in peripheral blood T cells during treatment, globally suggesting a therapeutic response (26). Similarly, the slight expression of PD-L1 in PT can be considered another favorable prognostic feature, although still debated (27–29). Despite the role of LAG-3 and TIM-3 is still open to discussion (30–34), these molecules may not be considered as independent and isolated immune inhibitors but rather as co-indicators together with PD-1 of a still active inflammatory response where T cells are activated to exert their antitumor activity, and a part of them is progressively turning off and becoming exhausted. Therefore, the co-expression of PD-1, LAG-3, and TIM-3 on T cells suggests that additional combinatory immune checkpoint blockade therapies could be required in poorly responding cases (35, 36).

In summary, we present an exceptional and clinical complex case of a patient with MSI-H mCRC with a 4-year complete durable response after receiving exclusively immunotherapy agents, i.e., the PD-1 inhibitor nivolumab and the CTLA-4 inhibitor ipilimumab, without any relevant adverse event. In this case, the high efficacy of immunotherapy combination in MSI-H mCRC also relied, from a pathological point of view, on the confirmation of a pCR, a rare event with standard therapies that is associated to long-term survival or even the chance for cure. Nevertheless, the extensive molecular analysis and immunoprofiling carried out provided additional dynamical insights about the evolution of an immune response, which ultimately leads to a therapeutic success. In addition, the characterization of the immune contexture in the primitive lesions, like the expression/co-expression of checkpoint inhibitors, can offer important predictive hints about the potential benefit of combinatorial immune checkpoint blockade therapies to overcome resistance in patients with poor response to primary treatments.

Overall, further studies are required to validate the current immunological conclusions and to identify patients in which surgery could be an option despite stage of disease or, conversely, when it could be avoided in the presence of complete response after immunotherapy treatment.

## Data availability statement

The original contributions presented in the study are included in the article/**Supplementary Material**. Further inquiries can be directed to the corresponding author.

## Ethics statement

Written informed consent was obtained from the individual(s) for the publication of any potentially identifiable images or data included in this article.

## Author contributions

SL, FB, FL, VZ, KC, and CG as medical doctors and Medical Oncology specialist were in charge of patient medical treatment; EG and UC performed surgical procedures; MF, ADT, and VA performed histopathology, immunophenotype and molecular assessment; GR and ARa performed radiological assessment; AT performed mIHC analysis; SDS and ADP performed cytofluorimetric analysis; SDS, AT, and ADP supervised and managed the data generation and data analysis; FC and SB performed the genomic and transcriptomic sequencing and the proteomic analyses; ARo contributed to the analysis and interpretation of the data and reviewed all drafts of the manuscript; all authors contribute to manuscript preparation, writing, revision and fully approved the content.
