# Supplementary figures and images for: Case report: Complete pathologic response with first-line immunotherapy combination in a young adult with massive liver dissemination of mismatch repair–deficient metastatic colorectal cancer: Immunological and molecular profiling
Source: Front Oncol. 2022 Dec 8;12:964219. doi: 10.3389/fonc.2022.964219 (PMC9791944; doi:10.3389/fonc.2022.964219)

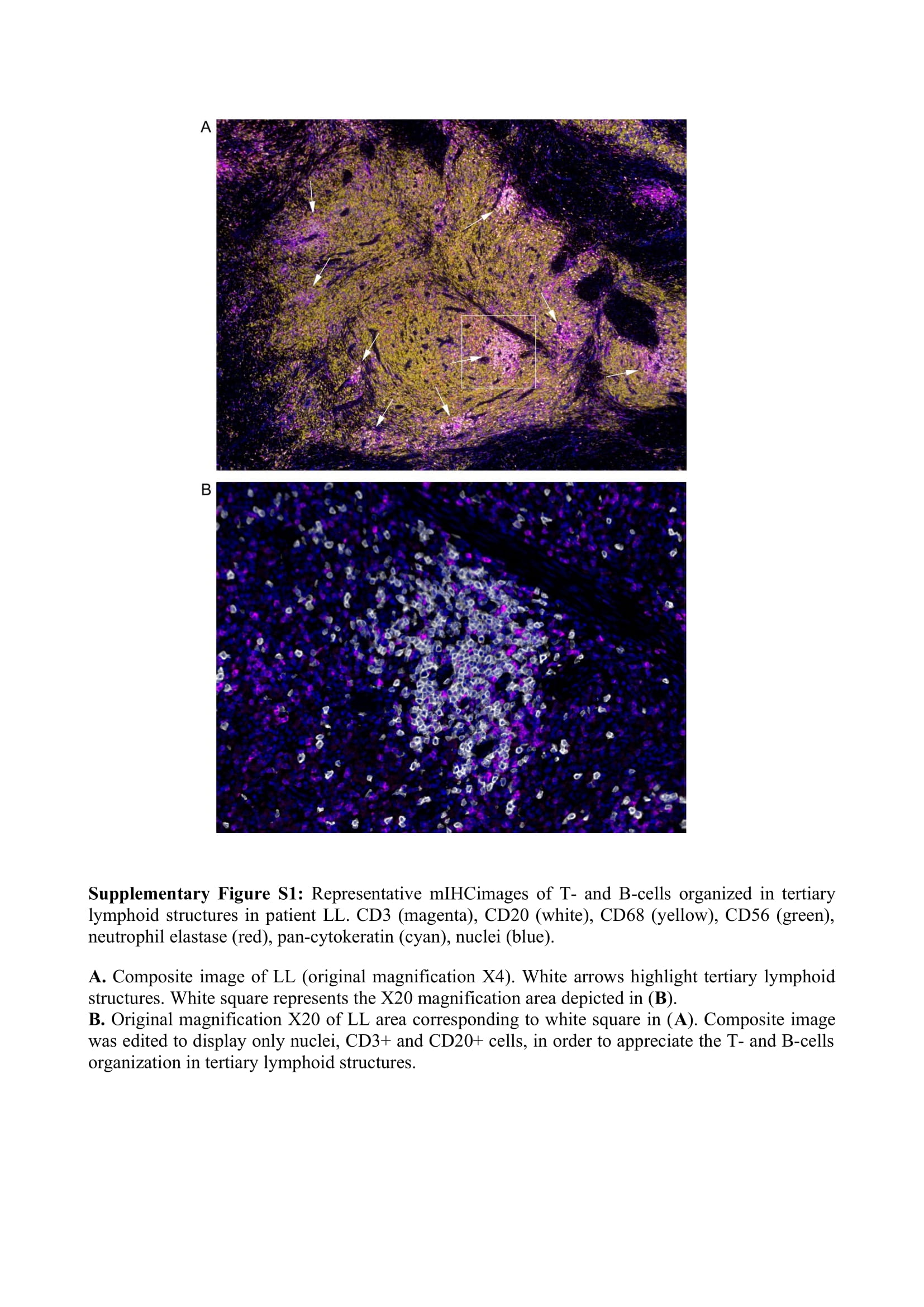

Supplement: Supplementary file 1 [file Image_1.jpeg]

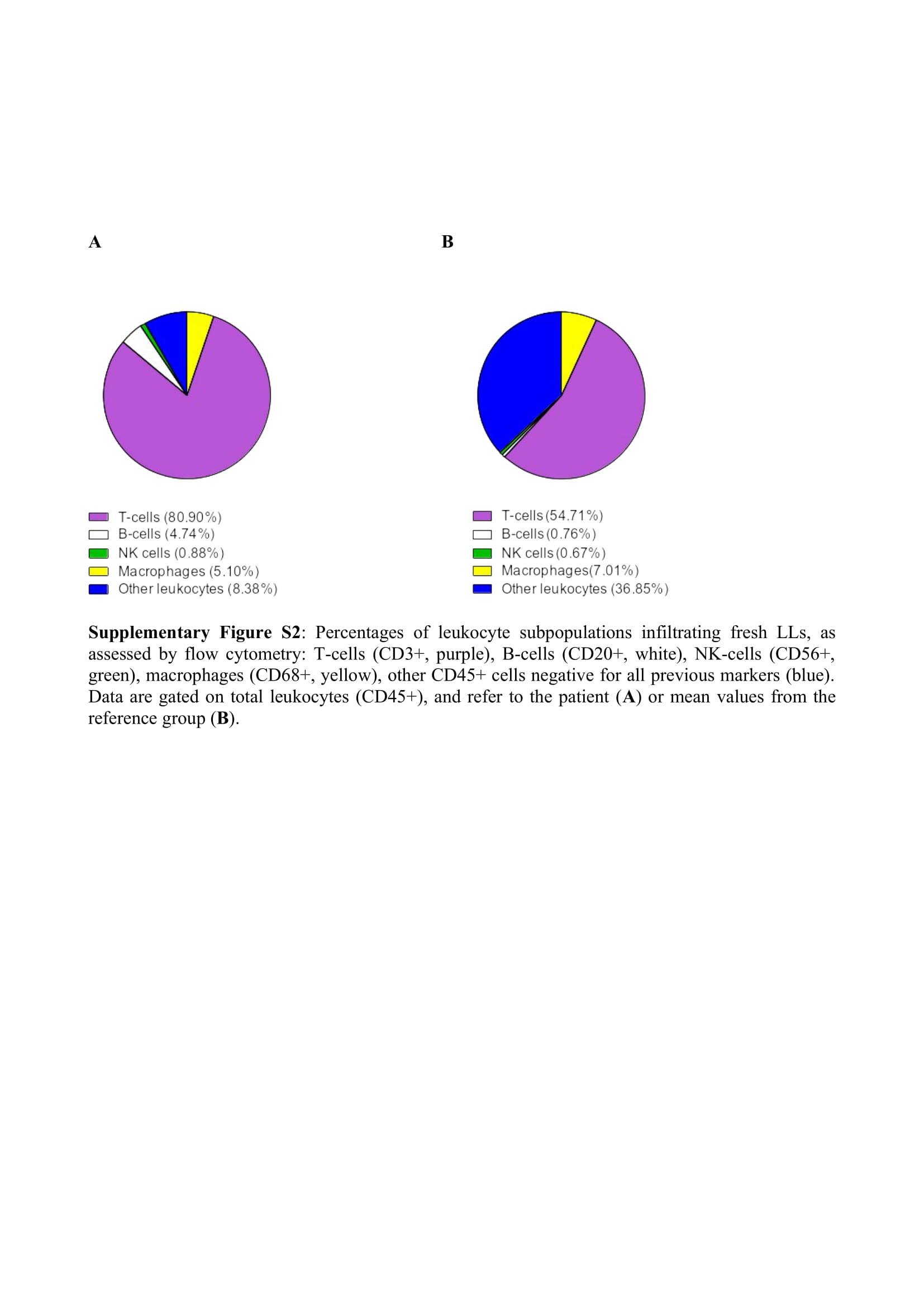

Supplement: Supplementary file 2 [file Image_2.jpeg]

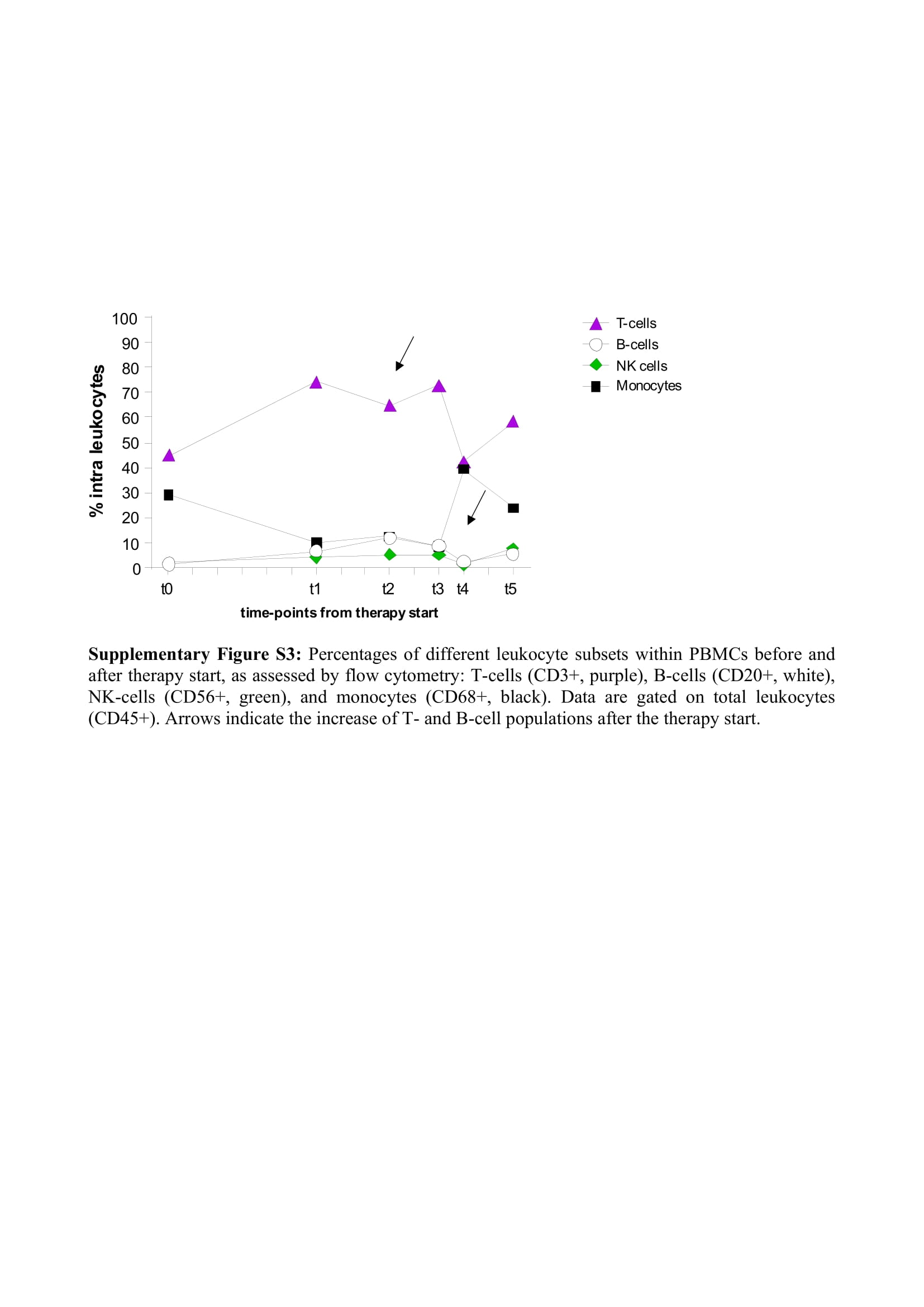

Supplement: Supplementary file 3 [file Image_3.jpeg]

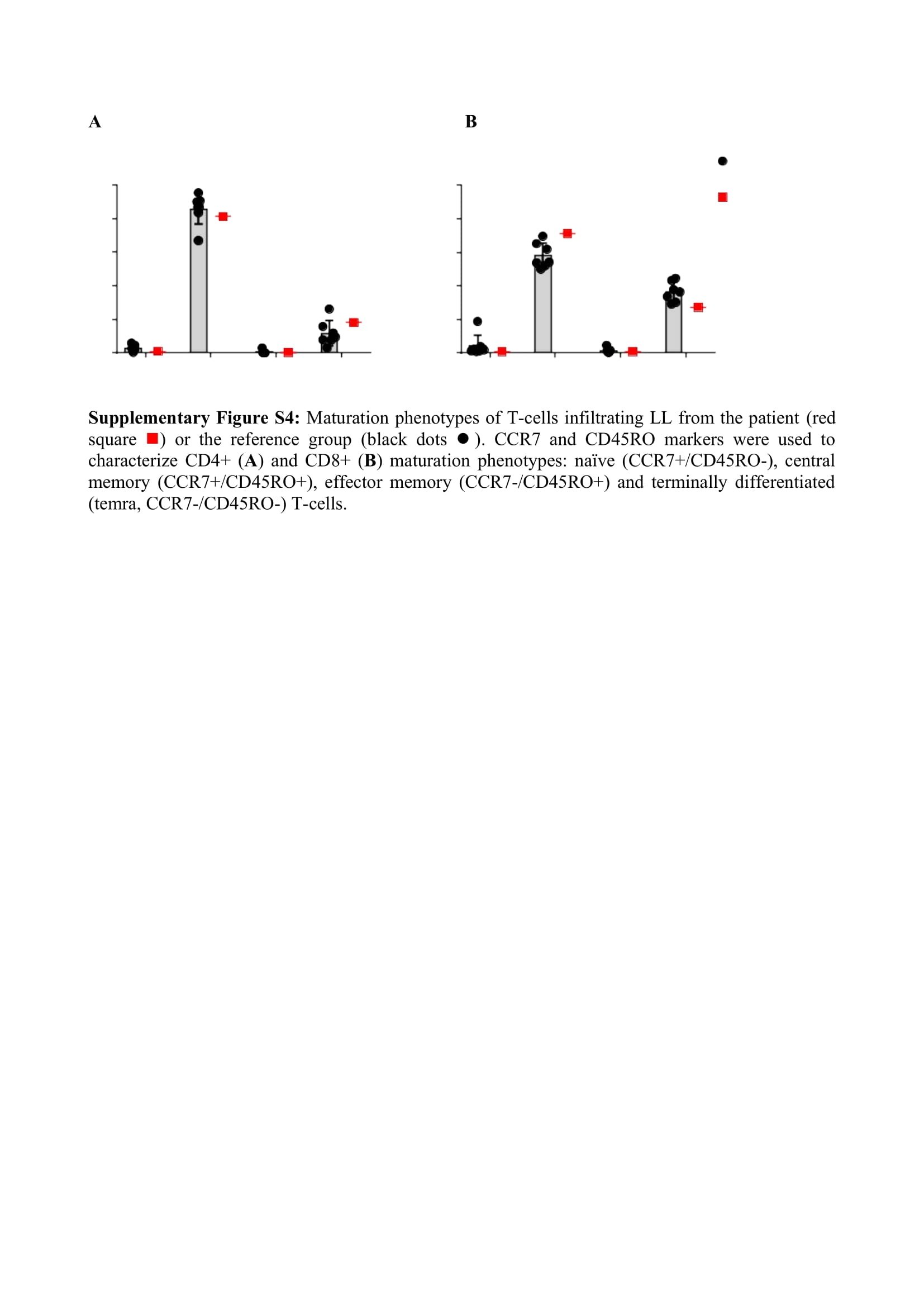

Supplement: Supplementary file 4 [file Image_4.jpeg]

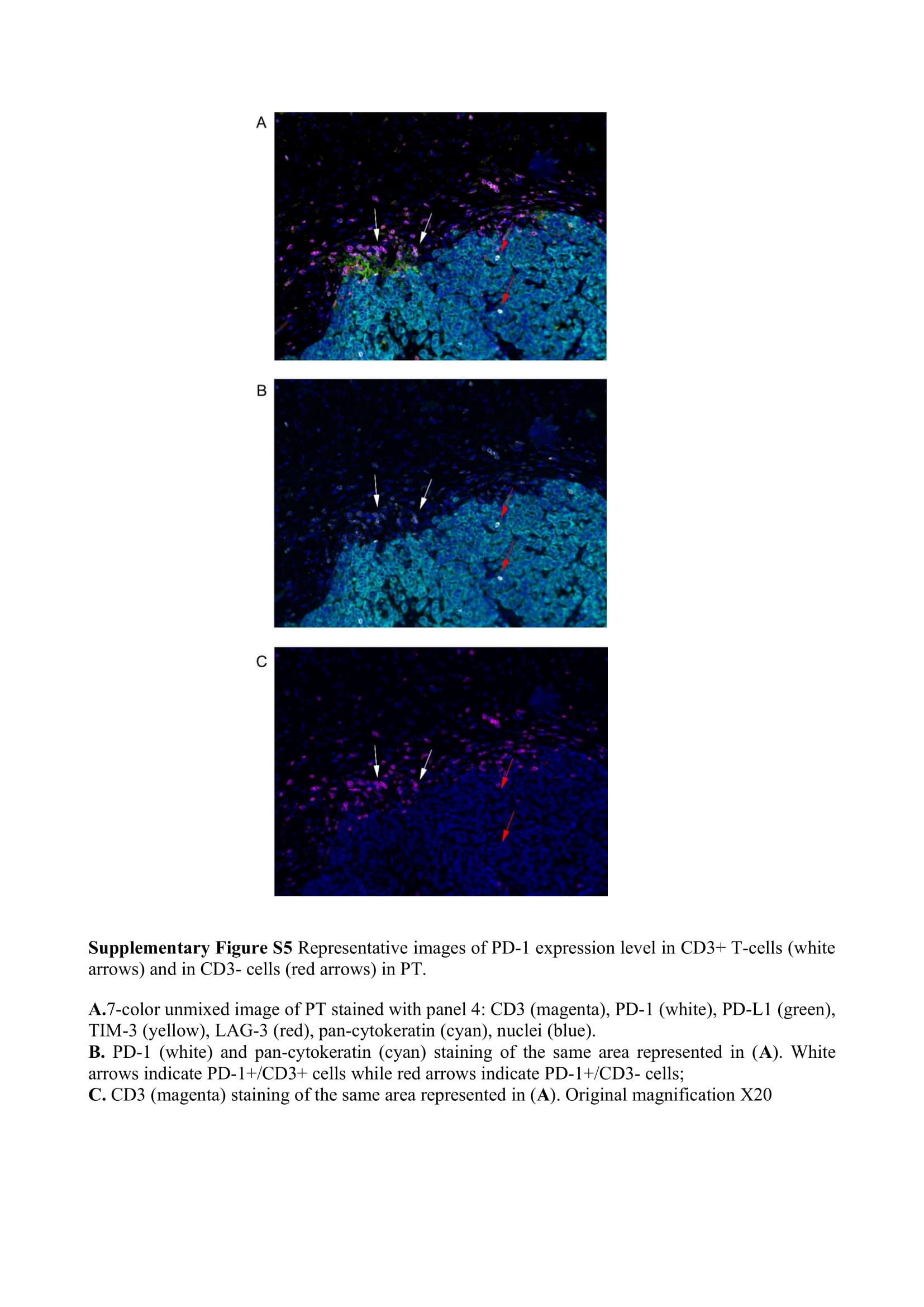

Supplement: Supplementary file 5 [file Image_5.jpeg]

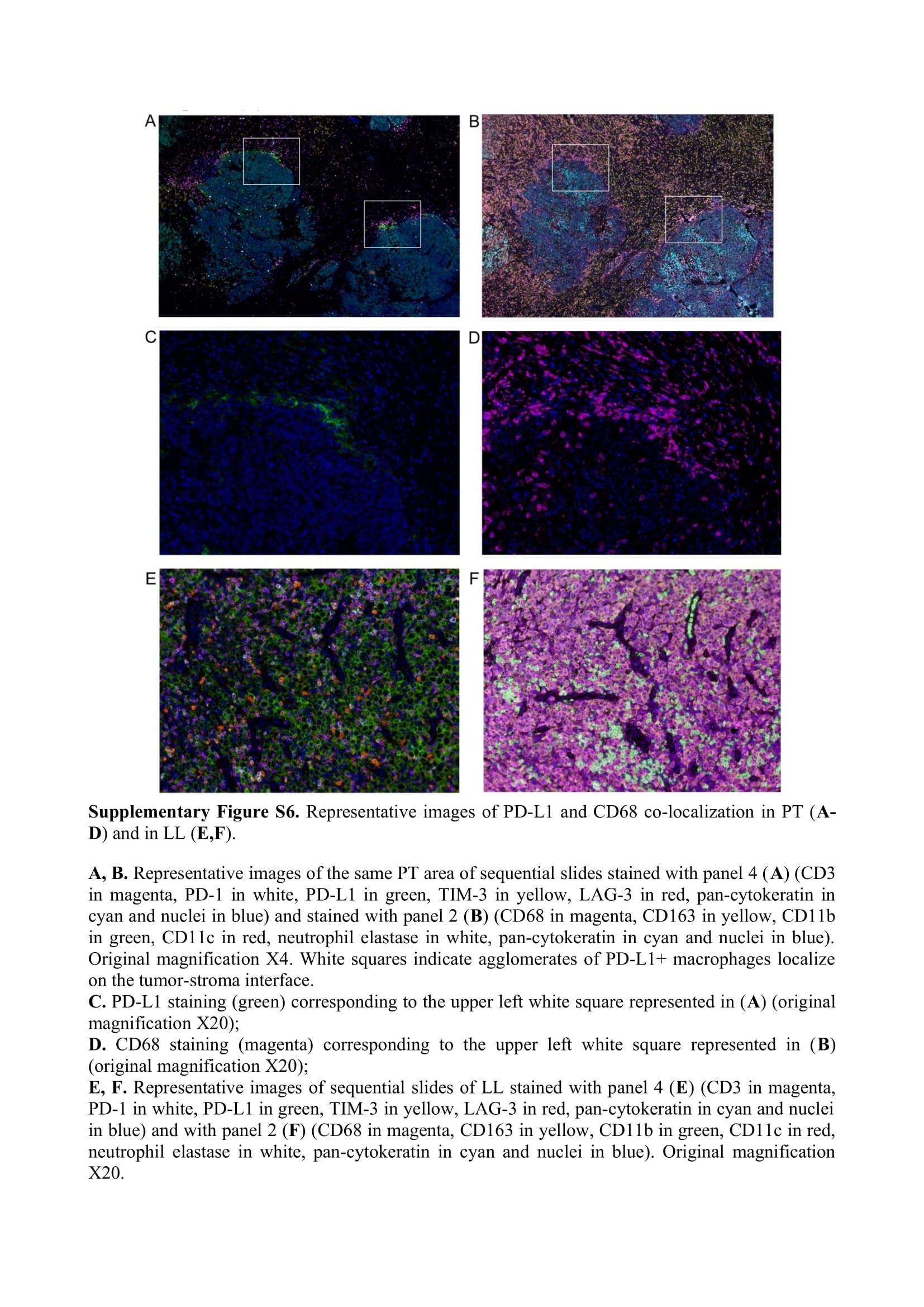

Supplement: Supplementary file 6 [file Image_6.jpeg]
