# Supplementary material for: Case report: Complete pathologic response with first-line immunotherapy combination in a young adult with massive liver dissemination of mismatch repair–deficient metastatic colorectal cancer: Immunological and molecular profiling
Source: Front Oncol. 2022 Dec 8;12:964219. doi: 10.3389/fonc.2022.964219 (PMC9791944; doi:10.3389/fonc.2022.964219)
Supplement: Supplementary file 7 [file Table_1.docx]

| Pts n | MSI status | KRAS status | NRAS status | BRAF status | Chemotherapy regimen |
| --- | --- | --- | --- | --- | --- |
| 1 | MSI-H | wt | wt | mut | XELOX |
| 2 | MSS | wt | wt | wt | XELOX |
| 3 | MSS | wt | wt | wt | FOLFIRI - Cetuximab |
| 4 | MSS | wt | wt | wt | FOLFOXIRI - Bevacizumab |
| 5 | MSS | wt | wt | wt | FOLFOX - Panitumumab |
| 6 | MSS | wt | wt | wt | FOLFOX - Panitumumab |

**Supplementary Table 1:** Molecular profile and chemotherapy regimen of the patients included in the reference group. All the patients underwent hepatic surgery after chemotherapy regimen specified below and histological samples of the resected liver metastases were analyzed.
